# Association Between Focused Assessment with Sonography in Trauma (FAST) Results, Transfusion, and Injury in Trauma: A Multicenter Retrospective Cohort Study

**DOI:** 10.1016/j.acepjo.2026.100487

**Published:** 2026-08-14

**Authors:** Lachlan Driver, Angel Castro, Jenna LaColla, Trent She, Curtis Xu, Kristin H. Dwyer, Nikolai Schnittke, Zachary Boivin, Eric Lieu, Joseph R. Pare

**Affiliations:** 1 Department of Emergency Medicine, Brown University Health, Providence, Rhode Island, USA; 2 Department of Emergency Medicine, UMass Memorial Health, Worcester, Massachusetts, USA; 3 Department of Emergency Medicine & Department of Anesthesiology and Critical Care Medicine, Johns Hopkins University School of Medicine, Baltimore, Maryland, USA; 4 Department of Emergency Medicine, Yale University, New Haven, Connecticut, USA; 5 Department of Emergency Medicine, Hartford Hospital, Hartford, Connecticut, USA; 6 Department of Emergency Medicine, Oregon Health & Science University, Portland, Oregon, USA

**Keywords:** FAST, trauma, blood transfusion, hemorrhage, internal injury

## Abstract

**Objectives:**

The Focused Assessment with Sonography in Trauma (FAST) is central to early trauma resuscitation, but the extent to which FAST findings provide information beyond physiologic and laboratory markers remains incompletely characterized in contemporary practice.

**Methods:**

We conducted a retrospective multicenter cohort study of adult trauma patients who underwent FAST at 4 Level I trauma centers between 2021 and 2023. The primary outcome was blood transfusion in the emergency department (ED). The secondary outcome was a composite of severe internal injury associated with potential hemorrhage (liver/spleen/kidney injury, hemoperitoneum, pelvic fracture, retroperitoneal hematoma, vascular injury, or hemothorax). Mixed-effects logistic regression was performed. The adjusted odds ratios (aORs) for FAST result were calculated controlling for patient-level variables.

**Results:**

Among the 2151 trauma patients undergoing FAST, 1792 (83.3%) were documented negative, 335 (15.6%) positive, and 24 (1.1%) indeterminate. Overall, 610 patients (28.4%) were transfused, including 418/1792 (23.3%) FAST-negative, 179/335 (53.4%) FAST-positive, and 13/24 (54.2%) indeterminate (*P* < .001). Severe injuries occurred in 675 patients (31.4%), including 445/1792 (24.8%) FAST-negative, 216/335 (64.5%) FAST-positive, and 14/24 (58.3%) indeterminate (*P* < .001). In adjusted models, FAST-positive and indeterminate examinations were independently associated with transfusion (positive adjusted odds ratio [aOR] 3.38, 95% CI 2.53 to 4.50; indeterminate aOR 4.11, 95% CI 1.58 to 10.72) and with severe internal injury (positive aOR 4.82, 95% CI 3.66 to 6.35; indeterminate aOR 4.57, 95% CI 1.81 to 11.57).

**Conclusions:**

Among trauma patients undergoing FAST, positive examinations were independently associated with ED transfusion and severe internal injury. Although limited by small numbers, indeterminate FAST examinations demonstrated similarly elevated risk and should not be considered reassuring; however, these findings should be interpreted cautiously. These findings support the use of FAST as an early risk stratification tool that complements clinical and physiologic assessment.

**The Bottom Line**

In 2151 trauma patients who underwent a Focused Assessment with Sonography in Trauma examination at 4 Level I trauma centers, positive examinations were linked to higher rates of blood transfusion and severe internal injury, even after accounting for vital signs and laboratory tests. Nearly two-thirds of patients with positive examinations had severe internal injuries, but one-quarter of patients with negative examinations also had clinically important injuries. Indeterminate examinations were uncommon but carried similar risks. These findings suggest that bedside ultrasound adds important information during trauma resuscitation and should be interpreted alongside the overall clinical picture.

## 1 Introduction

### 1.1 Background

Trauma is a leading cause of morbidity and mortality worldwide, and in the United States it is the fourth leading cause of death overall and the leading cause of death among individuals younger than 45 years.^1^^,^^2^ Among fatalities, uncontrolled bleeding is the most preventable cause of early death, with approximately 40% of trauma-related mortality caused by hemorrhage, particularly within the first hour of injury.^3^ The Focused Assessment with Sonography in Trauma (FAST) is widely used in early trauma resuscitation to rapidly detect free intraperitoneal/intrathoracic fluid at the bedside.^4^, ^5^, ^6^ Despite its integration into Advanced Trauma Life Support pathways, its value in modern trauma care—where computed tomography (CT) and laboratory markers are routinely incorporated—remains uncertain.^4^^,^^6^^,^^7^

### 1.2 Importance

Limited data exist on the association between FAST exam findings and blood transfusion,^8^ focusing on massive transfusion protocols^9^ or prehospital transfusions.^10^ Prior studies have suggested that positive FAST examinations are associated with more severe injury burden and higher rates of operative intervention and mortality.^11^ However, these data tend to derive from single-center cohorts, older data sets, or analyses that did not adjust for hemodynamic variables, lactate, or base deficit.^6^^,^^7^^,^^12^ Furthermore, the clinical implications of indeterminate FAST examinations are less well characterized, and the extent to which substantial internal injury may be present despite a negative FAST is a persistent concern at the bedside, particularly given the variable sensitivity of FAST.^4^^,^^13^^,^^14^

### 1.3 Goals of This Investigation

Although prior studies have demonstrated associations between FAST findings and transfusion requirements, many were conducted at single centers or used older datasets. We sought to evaluate whether FAST category (negative, positive, or indeterminate) was independently associated with early emergency department (ED) transfusion after adjustment for physiologic and laboratory markers of hemorrhagic shock in a contemporary multicenter cohort. As a secondary objective, we examined the association between FAST category and severe internal injury patterns associated with hemorrhage and potential need for transfusion or operative intervention.

## 2 Methods

### 2.1 Study design

We conducted a multicenter retrospective cohort study of patients evaluated at 4 EDs between January 1, 2021 and December 31, 2023. All participating hospitals were urban academic Level I trauma centers with active emergency medicine, ultrasound, and acute care surgical training programs and established incorporation of FAST into trauma resuscitation, and all have over 2000 trauma admissions annually. FAST examinations were performed and interpreted as part of routine clinical care at the discretion of treating clinicians. Although all centers routinely incorporated FAST into trauma evaluation, local training practices, reporting systems, and quality assurance processes varied across sites. Institutional review boards at participating sites approved the study with waiver of informed consent. This study is reported in accordance with POCUS as well as the STrengthening the Reporting of OBservational studies in Epidemiology (STROBE) guidelines for cohort studies.^15^^,^^16^

### 2.2 Selection of Participants

**Figure 1:**
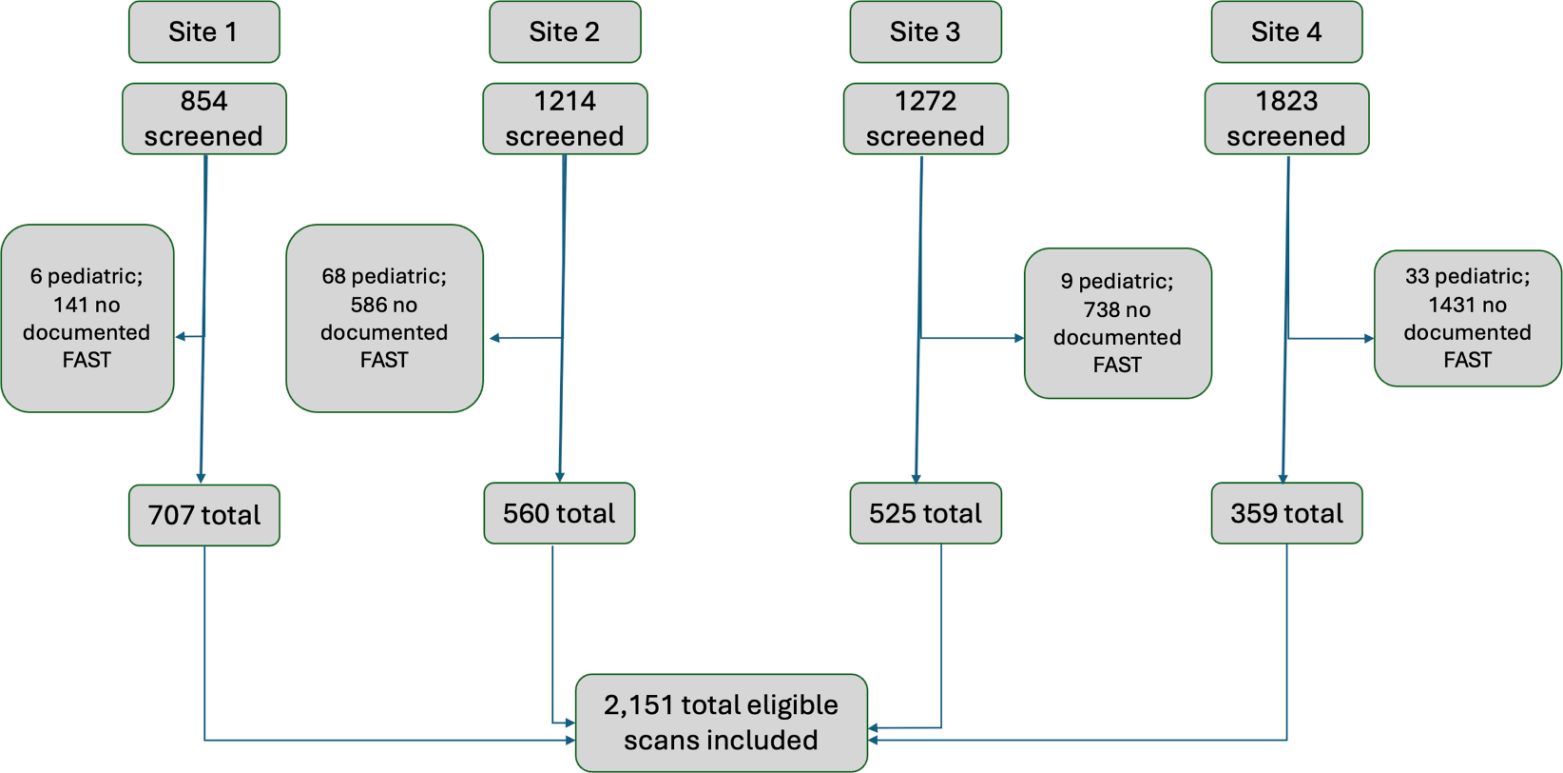
Flow chart of patients included for analysis.

Eligible encounters included adult trauma activations with a documented FAST during ED evaluation, identified by electronic health record (EHR) query. Trauma activation criteria were site-specific and previously published.^17^ We excluded patients <18 and encounters with missing FAST results, as shown in Fig 1. Encounters were analyzed independently. Outcomes were assessed during the index ED visit.

### 2.3 FAST Protocol and Definitions

FAST examinations were performed by emergency medicine physicians, advanced practice providers, or members of the trauma team, according to local practice. Standard FAST views included the right upper quadrant, left upper quadrant, pelvic, and subxiphoid windows. FAST category was defined using the original clinician-documented interpretation recorded in the medical record or ultrasound reporting system. We did not perform image re-review. We accounted for between-site practice differences by including site as a random intercept.

FAST results were categorized into 3 groups. Examinations documented as having no free fluid in the abdomen, chest, pelvis, or pericardial space were classified as negative. Examinations interpreted as demonstrating free fluid were classified as positive. Studies documented as equivocal, indeterminate, or technically limited were grouped as indeterminate. Pneumothorax findings were not incorporated because outcomes were hemorrhage-focused and thoracic air documentation was inconsistent across sites.

### 2.4 Data Collection and Variables

We abstracted demographics, mechanism, physiologic variables, laboratory values, imaging, and outcomes from the EHR. Age, weight, and key physiologic and laboratory variables were categorized *a priori* using clinically meaningful thresholds to improve interpretability and to accommodate nonlinear relationships and missingness.

Physiologic variables included lowest systolic blood pressure (SBP) and highest HR documented in the ED, as well as highest shock index (HR divided by SBP). For primary analyses, lowest SBP was categorized as <90 mm Hg vs ≥90 mm Hg, or not obtained. HR was categorized as ≥100 beats per minute vs <100 beats per minute, and shock index was categorized as <1, 1 to 2, or ≥2, or not obtained. Prehospital hypotension was defined as any prehospital SBP <90 mm Hg and was categorized as yes or no, or not obtained.

Laboratory data included the highest lactate value and bicarbonate deficit. Lactate was categorized as <2 mmol/L, 2 to 4 mmol/L, >4 mmol/L, or not obtained. Bicarbonate deficit was defined as HCO_3_ <20 mmol/L (≥4 mmol/L below reference 24 mmol/L) and categorized as present, absent, or not obtained. For key covariates (SBP, heart rate, shock index, lactate, bicarbonate deficit, and prehospital hypotension), missingness was handled using explicit “not obtained” indicator categories to preserve cohort size and reflect real-world significance in trauma patient care. Mechanism of injury was categorized as blunt or penetrating.

### 2.5 Outcomes

The primary outcome was blood product transfusion during the index ED stay, defined as administration of packed red blood cells or whole blood prior to ED departure. Timing of transfusion relative to FAST completion could not always be determined retrospectively.

The secondary outcome was the presence of severe internal injury that may require transfusion or operative intervention. We defined this outcome *a priori* as a composite endpoint consisting of injuries associated with hemorrhage and potential escalation of care. Severe internal injury was defined as any of the following: solid organ injury to the spleen, liver, or kidney; hemoperitoneum; pelvic fracture; retroperitoneal hematoma; major vascular injury (including injuries to the aorta, inferior vena cava, or iliac vessels); or hemothorax. These findings were ascertained primarily from CT imaging reports and operative notes. In patients who died before definitive imaging or operative intervention, ED documentation was used to capture clinically apparent injuries. For individual injury components, values were coded as present, absent, or not documented. The composite “severe internal injury” indicator was coded as present if any component was documented present. Severe internal injury was analyzed as a binary outcome using this composite definition.

### 2.6 Primary Data Analysis

Cohort characteristics were reported with descriptive statistics. Baseline demographics, physiologic variables, and injury characteristics were described overall and then stratified by FAST result. Chi-squared tests were used to examine unadjusted associations between FAST category and each outcome.

To evaluate the association between FAST result and the primary outcome of ED transfusion, we constructed a mixed-effects logistic regression model with FAST category (negative, positive, indeterminate) as the primary independent variable. Covariates included age category, sex, weight category, SBP category, HR category, shock index category, mechanism (blunt vs penetrating), lactate category, bicarbonate deficit category, and prehospital hypotension. The ED site was included as a random effect to account for clustering. Models were fit using mixed-effects logistic regression with ED site included as a random intercept only to account for clustering by site; no random slopes were specified. Adjusted odds ratios with 95% CIs and *P* values were reported.

A second mixed-effects logistic regression model with the same covariates and random effect structure was constructed for the secondary outcome of severe internal injury. Statistical significance was defined as a two-sided *P* value < .05. All analyses were conducted in R version 4.5.0.^18^

To reflect clinical practice, missing values for key covariates (SBP, HR, shock index, lactate, bicarbonate deficit, prehospital hypotension) were handled using “not obtained” indicator categories. We assessed multicollinearity using generalized variance inflation factors in R^18^, which were all reassuring against multicollinearity. We performed a sensitivity analysis restricted to blunt trauma patients, repeating the mixed-effects model for ED transfusion.

## 3 Results

### 3.1 Cohort Characteristics

A total of 2151 trauma patients undergoing FAST in the ED met inclusion. Of these, 1792 (83.3%) had a negative FAST, 335 (15.6%) had a positive FAST, and 24 (1.1%) had an indeterminate FAST. Overall, 610 patients (28.4%) received packed red blood cells or whole blood in the ED. Baseline demographic, physiologic, and injury characteristics by FAST category are summarized in Table 1.

**Table 1:** Cohort characteristics by FAST category (negative/indeterminate/positive).

Characteristic	Overall	Negative	Positive	Indeterminate	*P* value^a^
	N = 2151 (%)	N = 1792 (%)	N = 335 (%)	N = 24 (%)	
Site number					<.001
1	707 (33)	609 (34)	89 (27)	9 (38)	
2	560 (26)	465 (26)	95 (28)	0 (0)	
3	525 (24)	404 (23)	106 (32)	15 (63)	
4	359 (17)	314 (18)	45 (13)	0 (0)	
Sex					.778
M	1468 (68)	1227 (68)	226 (67)	15 (63)	
F	683 (32)	565 (32)	109 (33)	9 (38)	
Age Groups (y)					.040
18-28	484 (23)	386 (22)	97 (29)	1 (4.2)	
29-39	430 (20)	354 (20)	68 (20)	8 (33)	
40-49	246 (11)	198 (11)	45 (13)	3 (13)	
50-59	268 (12)	227 (13)	38 (11)	3 (13)	
60-69	272 (13)	236 (13)	33 (9.9)	3 (13)	
70-79	219 (10)	188 (10)	28 (8.4)	3 (13)	
80+	232 (11)	203 (11)	26 (7.8)	3 (13)	
Race					.078
White	1178 (55)	988 (55)	175 (52)	15 (63)	
Black or African American	555 (26)	464 (26)	88 (26)	3 (13)	
Asian	30 (1.4)	19 (1.1)	10 (3.0)	1 (4.2)	
Other	388 (18)	321 (18)	62 (19)	5 (21)	
Mechanism					<.001
Blunt	1678 (78)	1421 (79)	236 (70)	21 (88)	
Penetrating	473 (22)	371 (21)	99 (30)	3 (13)	
Glasgow Coma Scale (GCS)					.126
3-8	370 (17)	296 (17)	65 (19)	9 (38)	
9-12	104 (4.8)	85 (4.7)	18 (5.4)	1 (4.2)	
13-15	1630 (76)	1,373 (77)	243 (73)	14 (58)	
Not documented	47 (2.2)	38 (2.1)	9 (2.7)	0 (0)	
Systolic blood pressure (mm Hg)					<.001
≥90	1328 (62)	1144 (64)	173 (52)	11 (46)	
<90	733 (34)	580 (32)	143 (43)	10 (42)	
Not documented	90 (4.2)	68 (3.8)	19 (5.7)	3 (13)	
Highest pulse					.029
<100	975 (45)	838 (47)	127 (38)	10 (42)	
≥100	1089 (51)	887 (49)	190 (57)	12 (50)	
Not documented	87 (4.0)	67 (3.7)	18 (5.4)	2 (8.3)	
Highest Shock Index					<.001
<1	1389 (65)	1209 (67)	169 (50)	11 (46)	
1-2	634 (29)	497 (28)	127 (38)	10 (42)	
≥2	47 (2.2)	31 (1.7)	16 (4.8)	0 (0)	
Not documented	81 (3.8)	55 (3.1)	23 (6.9)	3 (13)	
Lactate					.024
<2	500 (23)	416 (23)	80 (24)	4 (17)	
2-4	501 (23)	418 (23)	74 (22)	9 (38)	
>4	396 (18)	305 (17)	84 (25)	7 (29)	
Not obtained	754 (35)	653 (36)	97 (29)	4 (17)	
Is bicarbonate ≤ 20 mmol/L					<.001
No	889 (41)	775 (43)	101 (30)	13 (54)	
Yes	614 (29)	481 (27)	123 (37)	10 (42)	
Not obtained	648 (30)	536 (30)	111 (33)	1 (4.2)	
Prehospital hypotension (SBP <90 mm Hg)					<.001
No	727 (34)	612 (34)	100 (30)	15 (63)	
Yes	587 (27)	483 (27)	95 (28)	9 (38)	
Not documented	837 (39)	697 (39)	140 (42)	0 (0)	

FAST, Focused Assessment with Sonography in Trauma.

a Pearson’s Chi-squared test; Fisher’s exact test, as applicable.

### 3.2 FAST and ED Transfusion

**Figure 2:**
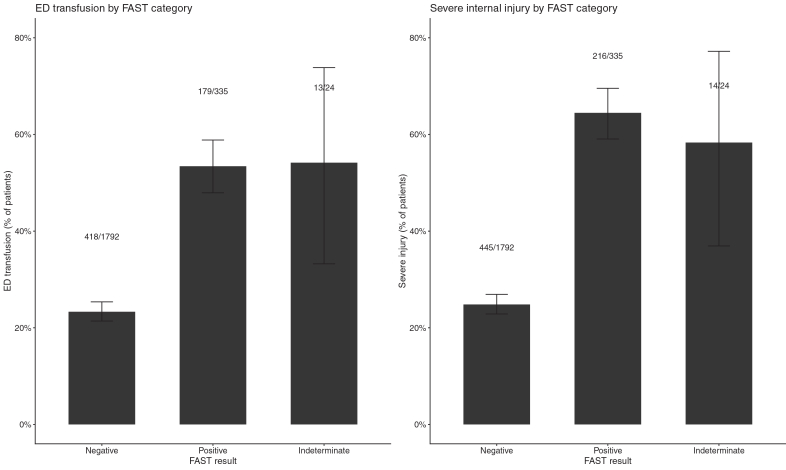
ED transfusion rate by FAST category (with 95% CIs) on the left, and severe internal injury prevalence by FAST category (with 95% CIs) on the right.

Transfusion rates differed significantly across FAST categories (Table 2, Fig 2), occurring in 418/1792 (23.3%) FAST-negative patients, 179/335 (53.4%) FAST-positive patients, and 13/24 (54.2%) patients with indeterminate examinations (*P* < .001).

**Table 2:** Outcomes by FAST category.

Characteristic	Overall N = 2151(%)	FAST negative (%)	FAST positive (%)	FAST indeterminate (%)	*P* value^a^
Transfused in the ED	610 (28)	418 (23)	179 (53)	13 (54)	<.001
Has severe internal injury	675 (31)	445 (25)	216 (64)	14 (58)	<.001

FAST, Focused Assessment with Sonography in Trauma; SBP, systolic blood pressure.

a Pearson’s Chi-squared test; Fisher’s exact test, as applicable.

**Figure 3:**
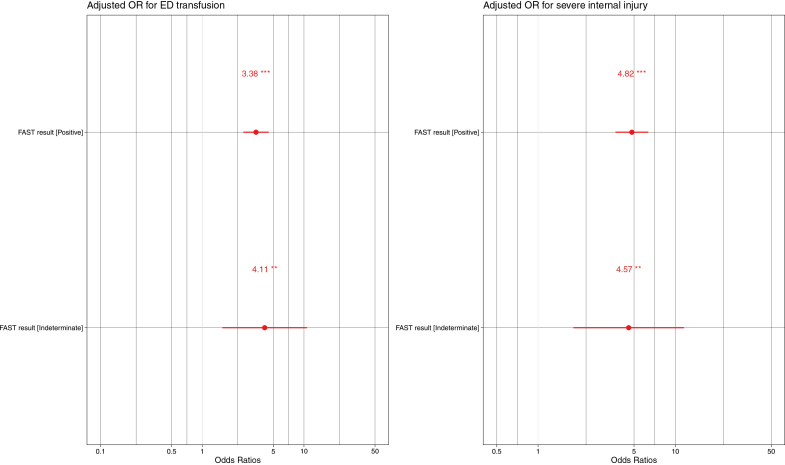
Adjusted Odds Ratios of Positive/Indeterminate FAST (compared with negative) on ED transfusion (right), as well as severe internal injury (left).

In the mixed-effects logistic regression model adjusted for age, sex, weight category, SBP, HR, shock index, mechanism of injury, lactate, bicarbonate deficit, prehospital hypotension, with ED site included as a random effect, a positive FAST remained strongly associated with ED transfusion compared with a negative FAST, as seen in Table 3. As in Fig 3, the adjusted odds ratio for transfusion among FAST-positive patients was 3.38 (95% CI 2.53 to 4.50; *P*< .001). Indeterminate FAST examinations also demonstrated significantly higher odds of transfusion relative to negative FAST, with an adjusted odds ratio of 4.11 (95% CI 1.58 to10.72; *P* = .004).

**Table 3:** Multivariable association between clinical variables and ED transfusion.

Variable	Adjusted OR	95% CI	*P* value
FAST result			
Positive vs negative	3.38	2.53-4.50	<.001
Indeterminate vs negative	4.11	1.58-10.72	.004
Age (y)^a^			
29-39 vs 18-28	1.12	0.78-1.61	.537
40-49	1.27	0.84-1.92	.257
50-59	1.22	0.81-1.84	.343
60-69	1.21	0.8-1.84	.37
70-79	0.79	0.50-1.24	.306
≥80	0.67	0.42-1.07	.092
Weight category (kg)^b^			
50-69 kg vs <50 kg	1.58	0.75-3.34	.234
70-89 kg	1.42	0.68-3.0	.354
90-109 kg	1.61	0.75-3.51	.223
≥110 kg	1.58	0.68-3.71	.286
Sex			
Female vs male	1.03	0.79-1.32	.848
ED heart rate (bpm)			
≥100 vs <100	1.12	0.86-1.45	.412
Missing	0.51	0.15-1.75	.18
Shock index			
1-2 vs <1	2.04	1.53-2.72	<.001
≥2 vs <1	3.18	1.47-6.87	.003
Not obtained	4.58	2.06-10.17	<.001
Mechanism			
Penetrating vs blunt	1.53	1.11-2.09	.009
ED systolic BP (mm Hg)			
<90 vs ≥90	4.68	3.56-6.17	<.001
Not obtained	1.47	0.42-5.1	.547
Lactate (mmol/L)			
2-4 vs <2	0.89	0.62-1.29	.541
>4 vs <2	1.75	1.19-2.57	.005
Not obtained	0.87	0.62-1.21	.394
Bicarbonate deficit			
≤−4 vs >−4	1.52	1.15-2.02	.004
Not obtained	1.05	0.48-2.31	.903
Prehospital hypotension (SBP <90 mm Hg)			
Present vs absent	1.90	1.39-2.61	<.001
Not recorded	3.54	2.12-5.92	<.001

FAST, Focused Assessment with Sonography in Trauma; SBP, systolic blood pressure.

Mixed-effects logistic regression with ED site as a random effect. Reference categories: FAST negative; weight <50 kg; SBP ≥90 mm Hg; HR <100 bpm; shock index <1; lactate <2 mmol/L; bicarbonate deficit >−4; no prehospital hypotension.

a Reference age category: 18-28 years.

b Reference weight category: <50 kg.

**Figure 4:**
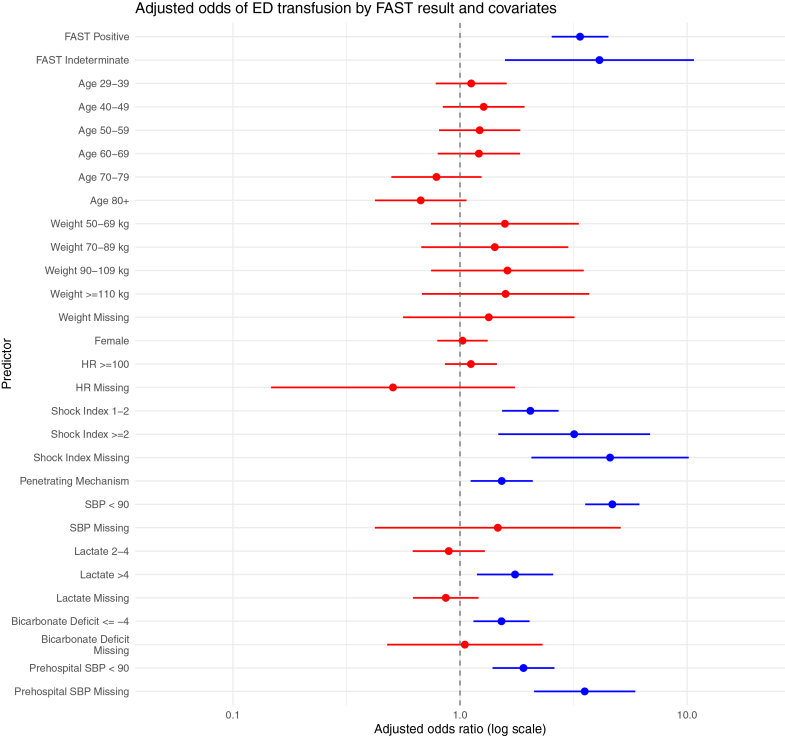
Adjusted odds of ED transfusion by FAST result and covariates.

Several physiologic and laboratory measures were also associated with transfusion, including hypotension, elevated shock index, elevated lactate, bicarbonate deficit, penetrating mechanism, and prehospital hypotension (Table 3, Fig 4).

### 3.3 FAST and Severe Internal Injury

Severe internal injury, defined as the composite outcome of solid organ injury, hemoperitoneum, pelvic fracture, retroperitoneal hematoma, major vascular injury, or hemothorax, was present in 675 of 2151 patients (31.4%). Rates varied substantially across FAST categories (Table 2, Fig 2). Among FAST-negative patients, 445 of 1792 (24.8%) had severe internal injury; the most common individual injury components were pelvic fracture (39.1%), vascular injury (26.3%), hemothorax (25.8%), and hemoperitoneum (23.8%). Individual injury components by FAST category are shown in Table S2.

**Figure 5:**
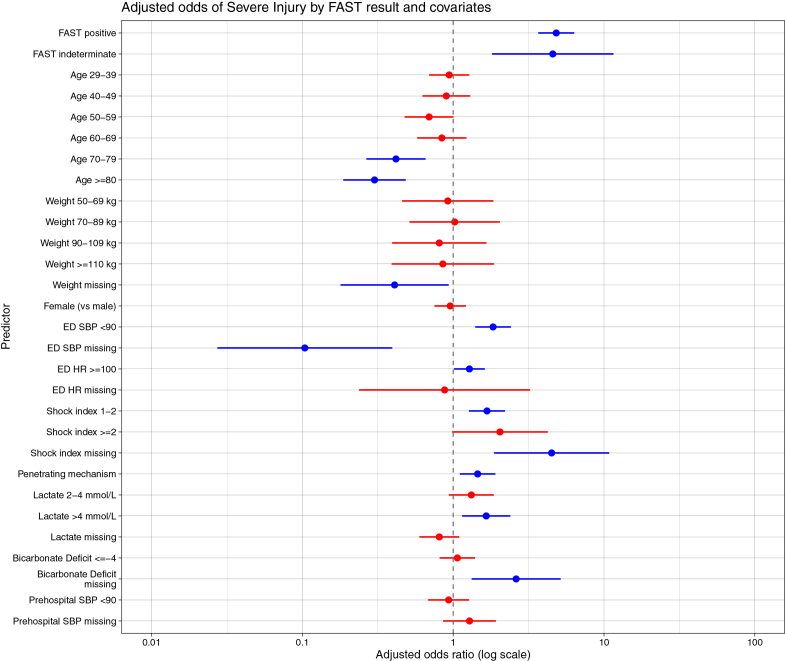
Adjusted odds of Severe Injury by FAST result and covariates.

Severe internal injury occurred in 445/1792 (24.8%) FAST-negative patients, 216/335 (64.5%) FAST-positive patients, and 14/24 (58.3%) patients with indeterminate examinations (*P* < .001). However, 119/335 (35.5%) FAST-positive patients and 10/24 (41.7%) patients with indeterminate examinations had no severe internal injury identified by the composite definition. In the multivariable mixed-effects model for severe internal injury, positive FAST remained a strong independent predictor after adjustment for covariates, as seen in Table 4 (Fig 4). As in Fig 5, compared with FAST-negative patients, those with a positive FAST had an adjusted odds ratio for severe internal injury of 4.82 (95% CI 3.66 to 6.35; *P* < .001). Indeterminate FAST was similarly associated with increased odds of severe internal injury (aOR 4.57; 95% CI 1.81 to 11.57; *P* = .001), although CIs were wider due to the small number of indeterminate studies. Several physiologic markers demonstrated similar directional associations as observed in the transfusion model.

**Table 4:** Multivariable association between clinical variables and severe internal injury.^a^

Variable	Adjusted OR	95% CI	*P* value
FAST result			
Positive vs negative	4.82	3.66-6.35	<.001
Indeterminate vs negative	4.57	1.81-11.57	.001
Age (y)^b^			
29-39 vs 18-28	0.94	0.69-1.28	.698
40-49	0.9	0.62-1.30	.573
50-59	0.69	0.48-1.01	.054
60-69	0.84	0.58-1.22	.367
70-79	0.42	0.27-0.66	<.001
≥80	0.30	0.19-0.49	<.001
Weight category^c^			
50-69 kg vs <50 kg	0.92	0.46-1.85	.816
70-89 kg	1.02	0.51-2.04	.948
90-109 kg	0.81	0.39-1.66	.564
≥110 kg	0.85	0.39-1.87	.692
Not recorded	0.41	0.18-0.93	.034
Sex			
Female vs male	0.96	0.75-1.22	.711
ED systolic BP (mm Hg)			
<90 vs ≥90	1.84	1.40-2.41	<.001
Not recorded	0.10	0.03-0.39	<0.001
ED heart rate (bpm)			
≥100 vs <100	1.28	1.01-1.62	.04
Not recorded	0.88	0.24-3.24	.844
Shock index			
1-2 vs <1	1.68	1.27-2.21	<.001
≥2 vs <1	2.04	0.98-4.24	.055
Not recorded	4.49	1.87-10.83	<.001
Mechanism			
Penetrating vs blunt	1.45	1.11-1.91	.007
Lactate (mmol/L)			
2-4 vs <2	1.32	0.94-1.86	.114
>4 vs <2	1.66	1.14-2.39	.007
Not obtained	0.81	0.60-1.10	.172
Bicarbonate deficit			
≤−4 vs >−4	1.07	0.81-1.40	.645
Not obtained	2.61	1.33-5.16	.006
Prehospital hypotension (SBP <90 mm Hg)			
Present vs absent	0.93	0.68-1.28	.664
Not documented	1.28	0.86-1.92	.225

FAST, Focused Assessment with Sonography in Trauma; SBP, systolic blood pressure.

a Severe internal injury composite: solid organ injury (spleen, liver, kidney), hemoperitoneum, pelvic fracture, retroperitoneal hematoma, major vascular injury, or hemothorax.

b Reference age category: 18-28 years.

c Reference weight category: <50 kg.

In a sensitivity analysis restricted to blunt trauma patients, associations between FAST category and ED transfusion were similar in direction and magnitude to those observed in the primary analysis (Table S1).

## 4 Limitations

This study has several limitations. First, its retrospective design introduces potential misclassification across sites. There was no image review, and previous studies have shown a variable rate in FAST examination archival.^17^^,^^19^ Reliance on contemporaneous clinician interpretation and documentation may have introduced misclassification due to operator variability and inconsistent documentation practices. Second, the cohort included only patients who underwent a documented FAST examination. Because FAST was performed at clinician discretion and according to local trauma activation practices, selection bias is possible, as patients receiving FAST may differ systematically from trauma patients who did not undergo FAST evaluation. Consequently, these findings should be interpreted within the population of trauma patients undergoing FAST rather than generalized to all trauma patients. Third, transfusion practices vary across institutions, and although the site was modeled as a random effect, residual practice-pattern differences may persist. Fourth, “severe internal injury” was defined by a composite outcome, which may not capture all clinically relevant hemorrhage patterns (eg, isolated long-bone hemorrhage) and may classify certain clinically important injuries differently depending on documentation and imaging.

As this was an observational study, our findings are susceptible to confounding. Treating teams were aware of FAST findings in real time, and clinician awareness of a positive FAST may have directly influenced transfusion decisions. Consequently, the observed association between FAST category and ED transfusion likely reflects both underlying injury severity and clinician responses to FAST findings. Therefore, these results should not be interpreted as demonstrating a causal effect of FAST on transfusion decisions or patient outcomes. Missingness likely reflects workflow and clinical status and therefore may not have been random. Finally, although indeterminate FAST showed significantly increased odds of transfusion and injury similar to a positive FAST, this finding is limited by the small sample size and large CIs.

## 5 Discussion

In this multicenter retrospective cohort of trauma patients undergoing FAST across 4 Level I trauma EDs, FAST result was strongly associated with both early transfusion and severe hemorrhage-associated internal injury patterns, even after adjustment for physiologic and biochemical markers. A positive FAST was independently associated with over threefold higher odds of ED transfusion and an almost fivefold higher odds of severe internal injury compared with a negative FAST. Indeterminate FAST examinations behaved similarly, with effect sizes comparable with positive FAST for both outcomes. Collectively, these findings suggest that FAST provides information complementary to physiologic markers and remains a useful component of early trauma risk stratification.

FAST appeared to provide information complementary to traditional physiologic markers. Whereas hypotension and elevated shock index reflect the physiologic consequences of hemorrhage, FAST identifies anatomic evidence of bleeding. Together, these findings suggest that FAST contributes additional prognostic information beyond routine physiologic assessment. These findings are consistent with those of Stralec et al,^10^ who demonstrated an association between positive prehospital FAST examinations and severe hemorrhage in a contemporary multicenter cohort. Unlike their study, we evaluated ED FAST findings while adjusting for physiologic and laboratory markers associated with hemorrhagic shock, allowing assessment of the independent association of FAST beyond these variables. We additionally characterized indeterminate examinations and highlighted the persistent risk of severe injury despite negative FAST findings.

An unexpected finding was that patients aged 70 to 79 and ≥80 years had substantially lower adjusted odds of meeting our composite outcome compared with those aged 18 to 28 (Table 4). We hypothesize this finding reflects differences in case mix and how trauma activations are triggered. At many trauma centers, guidelines specify a lower threshold for trauma team activation in older patients (eg, age-based criteria for ground-level falls or minor mechanisms). As a result, older adults in our cohort may have been over-represented among lower-energy mechanisms and among activations without major intra-abdominal or pelvic injury, while still being at high risk for other serious pathology (eg, intracranial hemorrhage, rib fractures, or medical shock) not included in the composite outcome.

A notable operational finding is that only about half of FAST-positive patients received transfusion in the ED, whereas 1 in 4 FAST-negative patients still received blood. This discordance underscores that transfusion is not a direct reflection of finding hemoperitoneum alone. Clinically, transfusion decisions reflect combined information about mechanism, suspected bleeding compartment, and institutional practices. Additionally, some patients with positive FAST examinations may have proceeded rapidly to operative intervention, with subsequent blood product administration occurring outside the ED and therefore not captured by the primary outcome. Importantly, FAST findings should be interpreted within the broader clinical context and alongside physiologic parameters rather than in isolation. Transfusion decisions reflect multiple factors beyond FAST findings alone, including mechanism, physiology, and institutional practices.

FAST positivity and indeterminacy were also strongly associated with severe internal injury patterns linked to hemorrhage and potential transfusion need. Importantly, FAST-negative examinations should not be viewed as entirely reassuring, as one-quarter of FAST-negative patients met criteria for severe internal injury despite a negative examination. These findings reinforce that FAST should be interpreted in conjunction with mechanism and physiologic findings and should not be used in isolation to exclude clinically significant injury. This aligns with clinical experience and prior work demonstrating that FAST has imperfect sensitivity, particularly for injuries that bleed into compartments less reliably detected by ultrasound (eg, retroperitoneum) or for early injury. Among patients with FAST-negative examinations who met the severe internal injury definition, hemoperitoneum was nonetheless present in 106 cases (23.8%), although interval bleeding between FAST examination and definitive imaging or operative intervention may have contributed to this discrepancy. These data reinforce that a negative FAST reduces, but does not eliminate risk, and should not be used in isolation to delay definitive imaging or escalation of care when physiology and mechanism remain concerning.

Indeterminate FAST examinations occur in trauma care (often secondary to body habitus or bowel gas), yet their implications are unclear. In our analysis, although few in number (N = 24), documented indeterminate results carried odds of transfusion and severe internal injury similar to positive FAST. Given the small sample size and wide CIs, these findings should be interpreted cautiously and considered hypothesis-generating. Nevertheless, clinicians may wish to maintain a heightened level of suspicion when confronted with indeterminate examinations.

A significant portion of FAST-positive patients did not meet severe internal injury criteria. Site-level review suggests the majority of these positive FASTs without severe internal injury reflect nontraumatic sources of fluid (eg, ascites), as well as nonindex targets (eg, pericardial effusion) rather than true errors. In older or medically complex patients, ascites is more common, and ultrasound may detect real fluid that is not traumatic hemorrhage. These findings suggest the need to emphasize clinical context and documentation to distinguish a positive FAST that represents hemoperitoneum from one that represents nontraumatic fluid. However, even when fluid is thought to be nontraumatic in origin, free fluid on FAST should trigger closer observation and expedited workup, since it identifies an abnormal intrathoracic or intra-abdominal finding in a high-risk patient. It is possible that true false-positive FASTs were secondary to anatomic mimics or lipliner artifacts.^20^ Additionally, in the FAST-negative patients with severe internal injury, nearly half of injuries were isolated to areas in which ultrasound has known limitations such as the retroperitoneum.

Overall, these results support FAST as a useful component of rapid triage in trauma care as part of a multimodal assessment. FAST provides rapid anatomic information that complements physiologic markers, enabling teams to more quickly identify patients at high risk for injury requiring transfusion. These findings support continued integration of FAST with physiologic and laboratory assessment during trauma resuscitation and highlight the persistent risk of severe injury despite a negative FAST examination. Prospective evaluation of FAST-informed resuscitation strategies is warranted.

In a large multicenter cohort of trauma patients undergoing FAST evaluation at 4 Level I trauma centers, FAST results were independently associated with both early transfusion and severe hemorrhage-associated injury patterns, even after adjustment for physiology, laboratory values, and mechanism. Negative FAST examinations did not exclude clinically significant injury. These findings support continued integration of FAST into early trauma resuscitation as a rapid risk stratification tool that complements physiologic assessment and helps guide timely escalation of care. Prospective studies are needed to determine whether FAST-informed resuscitation strategies improve clinical outcomes.

## Author Contributions

LD and JRP: conception and study design. LD, KHD, and JRP: literature review. LD, AC, JL, TS, CX, KHD, NS, ZB, EL, and JRP: data acquisition. LD and JRP: data analysis and interpretation. LD and KHD: drafting of the manuscript. LD, AC, JL, TS, CX, KHD, NS, ZB, EL, and JRP: critical revision.

## Funding and Support

By *JACEP Open* policy, all authors are required to disclose any and all commercial, financial, and other relationships in any way related to the subject of this article as per ICMJE conflict of interest guidelines (see www.icmje.org). This study received no external funding.

## Conflicts of Interest

LD, AC, JL, TS, CX, KHD, ZB, EL, and JRP have affirmed they have no conflicts of interest to declare. NS reports research support from BARDA, GE, and Philips, which is not related to the current study.
