# Supplementary material for: Association Between Focused Assessment with Sonography in Trauma (FAST) Results, Transfusion, and Injury in Trauma: A Multicenter Retrospective Cohort Study
Source: J Am Coll Emerg Physicians Open. 2026 Aug 14;7(5):100487. doi: 10.1016/j.acepjo.2026.100487 (PMC13482603; doi:10.1016/j.acepjo.2026.100487)
Supplement: Supplementary file 1 [file mmc1.docx]

**Supplementary Table 1: Blunt trauma sensitivity analysis: multivariable association with ED transfusion**

| **Variable** | **Adjusted Odds Ratio** | **95% CI** | **p-value** |
| --- | --- | --- | --- |
| FAST result: Positive (vs Negative) | 3.57 | 2.54–5.01 | <0.001 |
| FAST result: Indeterminate (vs Negative) | 4.22 | 1.49–11.94 | 0.007 |
| Age 29–39 (vs 18–28) | 1.04 | 0.66–1.64 | 0.858 |
| Age 40–49 (vs 18–28) | 1.14 | 0.68–1.89 | 0.621 |
| Age 50–59 (vs 18–28) | 1.2 | 0.76–1.90 | 0.44 |
| Age 60–69 (vs 18–28) | 1.09 | 0.69–1.71 | 0.708 |
| Age 70–79 (vs 18–28) | 0.72 | 0.44–1.18 | 0.194 |
| Age ≥80 (vs 18–28) | 0.65 | 0.40–1.06 | 0.082 |
| Weight 50–69 kg (vs <50 kg) | 1.87 | 0.86–4.05 | 0.114 |
| Weight 70–89 kg (vs <50 kg) | 1.33 | 0.61–2.88 | 0.471 |
| Weight 90–109 kg (vs <50 kg) | 1.67 | 0.74–3.75 | 0.214 |
| Weight ≥110 kg (vs <50 kg) | 2.27 | 0.93–5.55 | 0.071 |
| Weight not documented (vs <50 kg) | 1.47 | 0.58–3.70 | 0.418 |
| Female (vs Male) | 0.98 | 0.75–1.29 | 0.894 |
| ED SBP <90 (vs ≥90) | 4.21 | 3.11–5.71 | <0.001 |
| ED SBP missing (vs ≥90) | 1.17 | 0.27–5.12 | 0.834 |
| ED HR ≥100 (vs <100) | 1.22 | 0.90–1.64 | 0.197 |
| ED HR not documented (vs <100) | 0.96 | 0.21–4.32 | 0.96 |
| Shock index 1–2 (vs <1) | 1.83 | 1.33–2.53 | <0.001 |
| Shock index ≥2 (vs <1) | 3.47 | 1.44–8.40 | 0.006 |
| Shock index not documented (vs <1) | 2.85 | 1.04–7.75 | 0.041 |
| Lactate 2–4 (vs <2) | 0.85 | 0.57–1.27 | 0.42 |
| Lactate >4 (vs <2) | 1.47 | 0.96–2.26 | 0.08 |
| Lactate not obtained (vs <2) | 0.94 | 0.64–1.37 | 0.732 |
| Bicarbonate deficit present (vs absent) | 1.59 | 1.16–2.19 | 0.004 |
| Bicarbonate not obtained (vs deficit absent) | 0.82 | 0.32–2.11 | 0.683 |
| Prehospital hypotension: Yes (vs No) | 2.02 | 1.44–2.85 | <0.001 |
| Prehospital hypotension: Not documented (vs No) | 4.4 | 2.35–8.25 | <0.001 |

Mixed-effects regression model restricted to blunt trauma patients. Reference categories: FAST negative; age 18–28 years; weight <50 kg; male sex; ED SBP ≥90 mmHg; ED HR <100 bpm; shock index <1; lactate <2 mmol/L; base deficit absent; no prehospital hypotension. “Missing” categories reflect not documented/not obtained values.
