# Supplementary material for: Association Between Focused Assessment with Sonography in Trauma (FAST) Results, Transfusion, and Injury in Trauma: A Multicenter Retrospective Cohort Study
Source: J Am Coll Emerg Physicians Open. 2026 Aug 14;7(5):100487. doi: 10.1016/j.acepjo.2026.100487 (PMC13482603; doi:10.1016/j.acepjo.2026.100487)
Supplement: Supplementary file 2 [file mmc2.docx]

**Supplementary Table 2. Individual Components of Severe Internal Injury by FAST Category**

| **Characteristic** | **Overall*^1^***  **N = 2,151** | **FAST negative*^1^*** | **FAST positive*^1^*** | **FAST indeterminate*^1^*** | **p-value*^2^*** |
| --- | --- | --- | --- | --- | --- |
| Splenic Injury |  |  |  |  | <0.001 |
| No | 1,893 (88%) | 1,640 (92%) | 236 (70%) | 17 (71%) |  |
| Yes | 139 (6.5%) | 55 (3.1%) | 80 (24%) | 4 (17%) |  |
| Not documented | 119 (5.5%) | 97 (5.4%) | 19 (5.7%) | 3 (13%) |  |
| Liver Injury |  |  |  |  | <0.001 |
| No | 1,887 (88%) | 1,625 (91%) | 245 (73%) | 17 (71%) |  |
| Yes | 144 (6.7%) | 70 (3.9%) | 70 (21%) | 4 (17%) |  |
| Not documented | 120 (5.6%) | 97 (5.4%) | 20 (6.0%) | 3 (13%) |  |
| Kidney Injury |  |  |  |  | <0.001 |
| No | 1,947 (91%) | 1,641 (92%) | 288 (86%) | 18 (75%) |  |
| Yes | 85 (4.0%) | 55 (3.1%) | 27 (8.1%) | 3 (13%) |  |
| Not documented | 119 (5.5%) | 96 (5.4%) | 20 (6.0%) | 3 (13%) |  |
| Hemoperitoneum |  |  |  |  | <0.001 |
| No | 1,782 (83%) | 1,588 (89%) | 176 (53%) | 18 (75%) |  |
| Yes | 251 (12%) | 106 (5.9%) | 141 (42%) | 4 (17%) |  |
| Not documented | 118 (5.5%) | 98 (5.5%) | 18 (5.4%) | 2 (8.3%) |  |
| Pelvic Injury |  |  |  |  | 0.002 |
| No | 1,805 (84%) | 1,519 (85%) | 273 (81%) | 13 (54%) |  |
| Yes | 223 (10%) | 174 (9.7%) | 41 (12%) | 8 (33%) |  |
| Not documented | 123 (5.7%) | 99 (5.5%) | 21 (6.3%) | 3 (13%) |  |
| RP hematoma |  |  |  |  | 0.001 |
| No | 1,919 (89%) | 1,616 (90%) | 284 (85%) | 19 (79%) |  |
| Yes | 113 (5.3%) | 79 (4.4%) | 32 (9.6%) | 2 (8.3%) |  |
| Not documented | 119 (5.5%) | 97 (5.4%) | 19 (5.7%) | 3 (13%) |  |
| Vascular Injury |  |  |  |  | <0.001 |
| No | 1,870 (87%) | 1,580 (88%) | 271 (81%) | 19 (79%) |  |
| Yes | 164 (7.6%) | 117 (6.5%) | 45 (13%) | 2 (8.3%) |  |
| Not documented | 117 (5.4%) | 95 (5.3%) | 19 (5.7%) | 3 (13%) |  |
| Hemothorax |  |  |  |  | <0.001 |
| No | 1,857 (86%) | 1,581 (88%) | 259 (77%) | 17 (71%) |  |
| Yes | 179 (8.3%) | 115 (6.4%) | 59 (18%) | 5 (21%) |  |
| Not documented | 115 (5.3%) | 96 (5.4%) | 17 (5.1%) | 2 (8.3%) |  |
| *^1^* n (%)  *^2^* Pearson’s Chi-squared test; Fisher’s exact test, as applicable |  |  |  |  |  |
